# Genetic variant of *Interleukin-18* gene is associated with the Frailty Index in the English Longitudinal Study of Ageing

**DOI:** 10.1093/ageing/afv122

**Published:** 2015-09-22

**Authors:** Krisztina Mekli, Alan Marshall, James Nazroo, Bram Vanhoutte, Neil Pendleton

**Affiliations:** 1 Cathie Marsh Institutefor Social Research, University of Manchester, Manchester M13 9PT, UK; 2 Institute of Brain, Behaviour and Mental Health, University of Manchester, Manchester, UK; 3 present address: School of Geography and Geosciences, University of St Andrews, St Andrews, UK

**Keywords:** genetics, SNP, Frailty Index, inflammation, older people

## Abstract

**Background:** the term frailty refers to a condition of increased vulnerability to stressors among older people, leading to a decline in homeostatic reserve. Frailty often leads to falls, hospitalisation and mortality, hence its importance for the delivery of health care to older adults. The pathophysiological mechanisms behind frailty are not well understood, but the decreased steroid-hormone production and elevated chronic systemic inflammation of older people appear to be major contributors.

**Method:** we used a sample of 3,160 individuals aged 50 or older from the English Longitudinal Study of Ageing and assessed their frailty status according to a Frailty Index. We selected 620 single nucleotide polymorphisms in genes involved in the steroid hormone or inflammatory pathways. We performed linear association analysis. The outcome variable was the square root transformation of the Frailty Index, with age and sex entered as covariates.

**Results:** the strongest signal was detected in the pro-inflammatory Interleukin-18 gene (rs360722, *P* = 0.0021, *β* = −0.015). Further significant signals were observed in the Interleukin-12 (rs4679868, *P* = 0.0062, *β* = −0.008 and rs9852519, *P* = 0.0077, *β* = −0.008), low density lipoprotein receptor-related protein 1 (rs1799986, *P* = 0.0065, *β* = 0.011) and Selectin-P (rs6131, *P* = 0.0097, *β* = −0.01) genes. None of these associations remain significant after Bonferroni correction.

**Conclusions:** we show potential associations between genetic variants of four genes and the frailty index. These genes are involved in the cholesterol transport and inflammatory pathway and, as such, our results provide further support for the involvement of the immunological processes in frailty of the elderly.

## Introduction

While it is accepted that the core feature of frailty is increased vulnerability to stressors due to impairments in multiple, inter-related systems that lead to decline in homeostatic reserve and resilience [1], the pathophysiological pathways behind this condition are not well understood.

Performance-based measures of frailty, such as the frailty phenotype [2] have found associations between decreased sex hormone levels such as testosterone, dehydroepiandrosterone (DHEA) and its sulphate derivative (DHEAS) and frailty [3, 4]. The levels of these hormones gradually decline with age, around 1–3%/year [5, 6]. The role that these hormones play in body mass [7, 8], muscle strength [9, 10] and bone mineral density [11, 12] gives biological support to the relation between these sex hormone levels and the performance-based measures of frailty.

Moreover, these hormones have an effect on inflammatory cytokines. Testosterone exerts a suppressive effect on pro-inflammatory cytokines, such as Interleukin-1β (IL-1β) and tumor necrosis factor (TNF) and increases anti-inflammatory cytokine Interleukin-10 (IL-10) [13], whereas DHEA seems to have an effect on Interleukin-6 (IL-6) levels [14].

In line with these results, age-related increases in cytokines and their association with performance measured frailty have been described in the literature [15, 16].

These results indicate a possible frailty mechanism, where the decreasing sex hormone and increasing inflammatory cytokine production during ageing yields a chronic low-level inflammatory state, which is responsible for the frailty symptoms.

Another robust and flexible measure of frailty is based on the concept of deficit accumulation quantified as a Frailty Index (FI) [17]. Deficits can be symptoms, or functional impairments that accumulate with age, such as problems carrying out daily activities, or chronic illnesses. Although this measure has received less attention in association studies, the FI was shown to be associated with lower levels of total and free testosterone and DHEAS in men [18] and increased levels of biomarkers of inflammation: basal IL-6, basal TNF and C-reactive protein [19].

The aim of this study was to conduct a candidate gene association study using the FI as a phenotypic measure in 3,160 community-dwelling individuals over the age of 50 in the English Longitudinal Study of Ageing (ELSA) panel study.

We selected genes involved in the steroid hormone biosynthesis and inflammation pathways, as evidence in the literature indicates their possible involvement in frailty pathophysiology (for example DHEAS [18], IL-6, TNF and C-reactive protein [19]). We hypothesised that single nucleotide polymorphisms (SNPs) within these genes, especially those ones which have been shown to affect the expression of their genes such as rs1800795 for *IL6* [20] and rs1800629 for *TNF* [21] will show significant association to FI.

## Methods

### Participants

The analyses are performed on a sample of 3,160 participants drawn from Wave 2 (2004) of the ELSA. Detailed description of ELSA can be found elsewhere ([22] www.elsa-project.ac.uk/, 14 September 2015, date last accessed). Briefly ELSA is a prospective panel study representative of community-dwelling men and women over 50 living in England. The participants have answered a computer assisted personal interview biannually collecting a wide range of information on social, psychological, functional and health domains. All participants provided signed consent and ethical approval was granted by the London Multi-Centre Research Ethics Committee.

### Genetic data

Genotype data of 620 SNPs for 3,160 participants were obtained from the publicly available ELSA DNA Repository (EDNAR).

Genotyping was performed by Illumina (San Diego, CA, USA) as part of a 1,536 Goldengate custom SNP panel using high-throughput BeadArrayTM technology.

### Phenotypic measures

An ELSA Frailty Index (FI) was created following guidance in the literature [17] using Wave 2 data. Briefly, the FI counts health-related problems (deficits) in a range of domains (activities of daily living, cognitive function, falls and fractures, joint replacement, vision, hearing, chronic diseases, cardiovascular diseases, depression). Only individuals with non-missing values for at least 30 of the 62 frailty components were included. A full list of the components is available in Appendix 1 (Supplementary data in *Age and Ageing* online). The FI had a negatively skewed distribution so we performed square root transformation.

### Statistical analysis

We used the Plink software for genetic association analyses [23]. Associations between genotypes and square root transformed FI were tested with linear regression using sex and age as covariates. Stata12 software (Stata Corporation, http://www.stata.com/, 14 September 2015, date last accessed) was used for the demographic and phenotypic analysis. Genetic power calculation was performed by Quanto software (http://biostats.usc.edu/software, 14 September 2015, date last accessed).

## Results

### Demographic and phenotypic results

Table 1 shows the demographic and phenotypic results. There were more females than males in the sample with no significant differences between the mean ages. The mean FI score was significantly higher in women than in men (two sample *t*-test, *P* < 0.0001).

**Table 1.** Demographic and phenotypic results

	Total sample	Males	Females
Number of participants	3,160	1,466	1,694
Mean age years (SE) in Wave 2	68.3 (0.10)	68.27 (0.14)	68.32 (0.14)
Mean FI scores (SE) in Wave 2	0.153 (0.0018)	0.140 (0.0025)	0.164 (0.0025)

SE, standard error; FI, Frailty Index.

**Figure 1.**
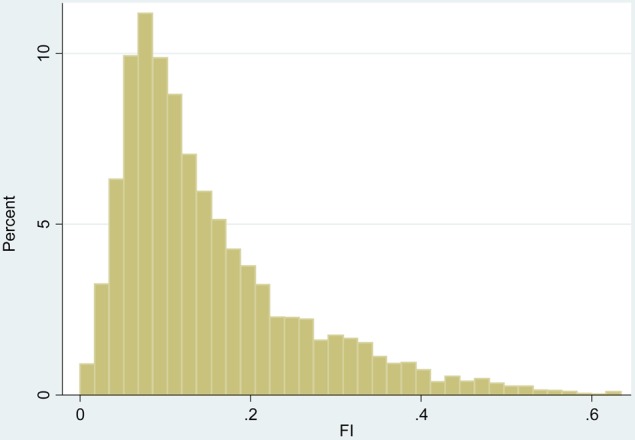
Frailty Index distribution in Wave 2 before square root transformation. FI, Frailty Index.

The distribution of the 62 item FI was skewed towards lower scores; therefore we performed square root transformation to normalise the distribution before the genetic association analysis (Figure 1). The lowest untransformed score was 0, the highest 0.634. As expected, age was positively associated with the FI which was lowest in the youngest group (age 60–64, mean FI = 0.133, SE = 0.003) and increased in the consecutive age groups (age 65–69, mean FI = 0.144, SE = 0.003, age 70–74, mean FI = 0.166, SE = 0.004, age 75–79, mean FI = 0.185, SE = 0.004).

### Genetic association analysis results

Of the received 620 SNPs, 15 were out of Hardy–Weinberg equilibrium (*P* < 0.01), and a further 15 had a minor allele frequency (MAF) below 5%, so these were excluded from the genetic association analysis, resulting in 590 SNPs used. The full list of 590 SNPs can be found in Appendix 2 (Supplementary data, available in *Age and Ageing* online).

The most significant results of the genetic association analysis can be seen in Table 2. One SNP reached significance level below *P* = 0.005, rs360722 (*IL18*), which decreased the FI (*β* = −0.015, uncorrected *P*-value =0.0021). Four SNPs were below the *P* = 0.01 threshold, rs4679868 (*IL12A*) (*β* = −0.008, uncorrected *P*-value = 0.0062), rs9852519 (*IL12A*) (*β* = −0.008, uncorrected *P*-value = 0.0077) and rs6131 (*SELP*) (*β* = −0.01, uncorrected *P*-value =0.0097), all decreasing the FI, whilst rs1799986 (*LRP1*) (*β* = 0.011, uncorrected *P*-value =0.0065) increased the FI. A further 12 SNPs were below the *P* = 0.05 significance level (see Supplementary data, Appendix 2, available in *Age and Ageing* online).

**Table 2.** Most significant results of genetic association analysis

SNP	Minor allele	MAF	*β*	*P*-value	Gene
rs360722	A	0.10	−0.015	0.0021	*IL-18*
rs4679868	A	0.38	−0.008	0.0062	*IL-12A*
rs1799986	A	0.15	0.011	0.0065	*LRP*
rs9852519	A	0.37	−0.008	0.0077	*IL-12A*
rs6131	A	0.16	−0.01	0.0097	*SELP*

SNP, small nucleotide polymorphism; MAF, minor allele frequency.

Several of these SNPs were in high linkage disequilibrium (rs4679868 and rs9852519, *r*^2^ = 0.92, rs2886666 and rs747825, *r*^2^ = 0.97 (all *IL12A*), rs6131 and rs3917729, *r*^2^ = 0.65, both *SELP*); therefore the number of independent signals is fewer.

None of these results survives the Bonferroni correction (*P* < 8.474E-05).

## Discussion

Our candidate gene association study is the first to investigate the association between the Frailty Index and genes involved in steroid hormone metabolism and inflammatory pathways.

Of the five top hits, inflammatory pathways are represented by four SNPs that are located in three genes, *IL18*, *IL12* and *SELP*. IL-18 is a powerful pro-inflammatory cytokine which plays a pivotal role in systemic and local inflammation, whereas IL12 is a major cytokine produced by antigen presenting cells and represents the link between the cellular and humoral branches of an effective host immune defence [24]. The third gene *SELP* encodes a cell adhesion molecule with a role in inflammation and haemostasis. During the former, it takes part in the tethering, rolling and weak adhesion of leukocytes, whereas in haemostasis it stabilises platelet aggregates and determines the size of aggregates [25].

IL-18 levels increase with age, similarly to IL-6 and CRP levels [26]. To our knowledge, IL-18 levels have not been investigated in relation to frailty, but higher IL-18 levels have been significantly associated with poorer physical performance (including balance, walk test and chair rise test) and with difficulties performing six activities of daily living (including bathing, dressing, eating, grooming, toileting and continence) [27]. Physical activity was also reported to be significantly and negatively associated with serum IL-18 levels in Japanese men [28]. The rs360722 A allele appeared to be protective against rheumatoid arthritis in another Japanese study [29]. This allele appeared to be protective in our study too, as it was associated with a decrease in the FI.

There is little information in the literature about the function of the other SNPs that we identified as potentially important. Rs4679868 A and rs9852519T alleles (both in *IL-12A*) have been associated with increased risk of primary biliary cirrhosis [30] whereas the A allele of rs6131 (*SELP*) has been found to lower platelet activation upon release of the cross-clamp in patients undergoing coronary artery bypass graft [31].

Naturally, these gene-products are all part of the very complex immune system and they are in interaction with several other molecules. For example, both IL18 and IL12 stimulate the interferon-γ production (IFN-γ) of Th1 cells, with a synergistic effect between them [24]. Moreover, rs360722 A allele (*IL18*) itself has been associated with a dose-dependent decrease in the secretion of the pro-inflammatory cytokine tumor necrosis factor (TNF) following smallpox vaccination [32].

It appears that SELP also has an effect on TNF. Adhesion of monocytes to SELP increased the secretion of TNF by the leukocytes when they were stimulated with platelet-activating factor *in vitro* [33].

Despite this, none of the eight SNPs in the *IFNG* and *TNF* gene regions showed an association with FI in our study. Other important inflammatory pathway genes, such as *CRP* or *IL6* (and its receptor *IL6R*), were also not associated with the FI, although CRP and IL6 levels have been associated with this phenotype [19]. One possible explanation for this is that IL-18 and IL-12 exert their effect on frailty independently from each other, not via increasing the IFN-γ or TNF levels, perhaps via pathways not related to IL6 or CRP. The inflammatory pathways consist of many members with complex interactions between them and it is not possible to evaluate these interactions fully within a candidate gene study of a limited number of genes.

It is important to note though, that as our results did not remain significant after the Bonferroni-correction; it is possible that they are false positive associations, although their agreement is compatible with some previous findings in the literature.

We also found a relationship below the *P* = 0.01 level for rs1799986, a synonymous variant located within the third exon of low density lipoprotein receptor-related protein 1 (*LRP1*) gene on chromosome 12. LRP1 is a large multiligand receptor involved in several cellular processes, including intracellular signalling, lipid homeostasis including cholesterol transport, and clearance of apoptotic cells [34] and thought to have a role in the pathology of Alzheimer disease (AD) [34, 35]. Analysis of post-mortem brain tissue showed that LRP1 levels normally decrease with ageing and were significantly lower in the brains of AD patients compared to controls [35]. There is contradictory evidence in the literature on the functionality of the rs1799986 polymorphism in AD [36, 37, 38]. Our results showed that the A allele appeared to increase the FI which is in agreement with a Spanish study, in which it was associated with increased risk of premature cardiovascular disease [39]. On the other hand, as rs1799986 is a synonymous mutation, it is possible that another SNP nearby is responsible for the effect. And this result did not remain statistically significant after correction for multiple testing.

This study has a number of limitations. First, only a proportion of the ELSA sample was genotyped, thus it may not be as representative as the full sample of the older adults in England. Secondly, the FI is based on self-reported questions which may introduce bias into the measure. The distribution of FI is skewed towards the lower end of the scale; therefore those with severe frailty are underrepresented in this sample of community-residing older people. This distribution however is in line with other reports from the literature. Thirdly, the genes and genotypic variants were selected from a publicly available dataset with limited genotyping, rather than specifically selected for the purpose of this study. Finally, we did not have the opportunity to measure serum IL-18 levels to corroborate our findings as data on this were not available in the dataset.

The strengths of our study include the derivation of the sample from a representative population study of older adults in England using a panel survey with standardised interviewer delivered validated protocols. Another important strength is the achieved sample size. The effective sample size required to test whether a gene main effect is associated with a continuous trait for 99% power, *α* = 0.05 (two-sided test), assuming outcomes that are modelled using linear regression (mean = 0.153, SD = 0.10, genetic effect = 0.01, MAF = 0.05) is 1,828, which is given in our study. As with all genetic association studies it would be advantageous to replicate our findings.

In summary, our candidate gene association study is the first one to investigate the genetic components of the Frailty Index and indicates the role of inflammation and cholesterol transport. Although the results are in line with some previous findings in the literature, none of the associations remains statistically significant after correction for multiple testing. This report generates hypotheses and hopefully will encourage further research into the molecular determinants of frailty in older adults.

**Key points**

- Limited evidence exists of plausible biological markers of frailty.
- To address this we conducted a candidate gene association study using the Frailty Index in the ELSA.
- The most significant signal was within *IL-18* gene with lesser associations in the *IL-12*, *LRP1* and *SELP* genes.
- Our findings support that inflammatory pathways are implicated in frailty.

## Conflicts of interest

None declared.

## Funding

This work was supported by the Medical Research Council (G1001375), UK. The funder had no further role in the conduct of the research.

## Supplementary data

Supplementary data mentioned in the text is available to subscribers in *Age and Ageing* online.

Supplementary Data
