# Supplementary material for: Genetic variant of Interleukin-18 gene is associated with the Frailty Index in the English Longitudinal Study of Ageing
Source: Age Ageing. 2015 Sep 22;44(6):938–42. doi: 10.1093/ageing/afv122 (PMC4621230; doi:10.1093/ageing/afv122)
Supplement: Supplementary Data [file supp_44_6_938__index.html]

Genetic variant of Interleukin-18 gene is associated with the Frailty Index in the English Longitudinal Study of Ageing — Supplementary Data 

# Genetic variant of *Interleukin-18* gene is associated with the Frailty Index in the English Longitudinal Study of Ageing

## Supplementary Data

Supplementary Data

- Supplementary Data - Docx file
