# Supplementary material for: Genetic variant of Interleukin-18 gene is associated with the Frailty Index in the English Longitudinal Study of Ageing
Source: Age Ageing. 2015 Sep 22;44(6):938–42. doi: 10.1093/ageing/afv122 (PMC4621230; doi:10.1093/ageing/afv122)
Supplement: Supplementary Data [file supp_afv122_afv122supp.docx]

SUPPLEMENTARY DATA

APPENDIX 1

| FI | **Type** | **Description** | **Values** |  |  |  |  |
| --- | --- | --- | --- | --- | --- | --- | --- |
| components | ADL: difficulties with activity due to health/physical problem | Difficulty with walking 100 yards | Not mentioned | Mentioned |  |  |  |
|  |  | Sitting for about two hours | Not mentioned | Mentioned |  |  |  |
|  |  | Getting up from a chair after sitting for long periods | Not mentioned | Mentioned |  |  |  |
|  |  | Climbing several flights of stairs without resting | Not mentioned | Mentioned |  |  |  |
|  |  | Climbing one flight of stairs without resting | Not mentioned | Mentioned |  |  |  |
|  |  | Stooping, kneeling, or crouching | Not mentioned | Mentioned |  |  |  |
|  |  | Reaching or extending your arms above shoulder level | Not mentioned | Mentioned |  |  |  |
|  |  | Pulling or pushing large objects like a living room chair | Not mentioned | Mentioned |  |  |  |
|  |  | Lifting or carrying weights over 10 pounds, like a heavy bag | Not mentioned | Mentioned |  |  |  |
|  |  | Picking up a 5p coin from a table | Not mentioned | Mentioned |  |  |  |
|  | IADL: difficulties with activity due to health/physical problem | Dressing, including putting on shoes and socks | Not mentioned | Mentioned |  |  |  |
|  |  | Walking across a room | Not mentioned | Mentioned |  |  |  |
|  |  | Bathing or showering | Not mentioned | Mentioned |  |  |  |
|  |  | Eating, such as cutting up your food | Not mentioned | Mentioned |  |  |  |
|  |  | Getting in or out of bed | Not mentioned | Mentioned |  |  |  |
|  |  | Using the toilet, including getting up or down | Not mentioned | Mentioned |  |  |  |
|  |  | Using a map to figure out how to get around in a strange place | Not mentioned | Mentioned |  |  |  |
|  |  | Preparing a hot meal | Not mentioned | Mentioned |  |  |  |
|  |  | Shopping for groceries | Not mentioned | Mentioned |  |  |  |
|  |  | Making telephone calls | Not mentioned | Mentioned |  |  |  |
|  |  | Taking medications | Not mentioned | Mentioned |  |  |  |
|  |  | Managing money, such as paying bills and keeping track of expenses | Not mentioned | Mentioned |  |  |  |
|  |  | Doing work around the house or garden | Not mentioned | Mentioned |  |  |  |
|  | General health | Self-reported general health | Excellent | Very good | Good | Fair | Poor |
|  | CES-D | Whether respondent has felt depressed much of the time during the past week | Good outcome | Bad outcome |  |  |  |
|  |  | Whether respondent felt everything they did during the past week was an effort | Good outcome | Bad outcome |  |  |  |
|  |  | Whether respondent felt their sleep was restless during the past week | Good outcome | Bad outcome |  |  |  |
|  |  | Whether respondent was happy much of the time during the past week | Good outcome | Bad outcome |  |  |  |
|  |  | Whether respondent felt lonely much of the time during the past week | Good outcome | Bad outcome |  |  |  |
|  |  | Whether respondent enjoyed life much of the time during the past week | Good outcome | Bad outcome |  |  |  |
|  |  | Whether respondent felt sad much of the time during the past week | Good outcome | Bad outcome |  |  |  |
|  |  | Whether respondent could not get going much of the time during the past week | Good outcome | Bad outcome |  |  |  |
|  | Diagnosed CVD | High blood pressure or hypertension | Not mentioned | Mentioned |  |  |  |
|  |  | Angina | Not mentioned | Mentioned |  |  |  |
|  |  | Heart attack (including myocardial infarction or coronary thrombosis) | Not mentioned | Mentioned |  |  |  |
|  |  | Congestive heart failure | Not mentioned | Mentioned |  |  |  |
|  |  | An abnormal heart rhythm | Not mentioned | Mentioned |  |  |  |
|  |  | Diabetes or high blood sugar | Not mentioned | Mentioned |  |  |  |
|  |  | A stroke (cerebral vascular disease) | Not mentioned | Mentioned |  |  |  |
|  | Diagnosed chronic condition | Chronic lung disease such as chronic bronchitis or emphysema | Not mentioned | Mentioned |  |  |  |
|  |  | Asthma | Not mentioned | Mentioned |  |  |  |
|  |  | Arthritis (including osteoarthritis , or rheumatism) | Not mentioned | Mentioned |  |  |  |
|  |  | Osteoporosis, sometimes called thin or brittle bones | Not mentioned | Mentioned |  |  |  |
|  |  | Cancer or a malignant tumour (excluding minor skin cancers) | Not mentioned | Mentioned |  |  |  |
|  |  | Parkinson's disease | Not mentioned | Mentioned |  |  |  |
|  |  | Any emotional, nervous or psychiatric problems | Not mentioned | Mentioned |  |  |  |
|  |  | Alzheimer's disease | Not mentioned | Mentioned |  |  |  |
|  |  | Dementia, organic brain syndrome, senility or any other serious memory impairment | Not mentioned | Mentioned |  |  |  |
|  | Eye sight | Self-reported eyesight (while using lenses, if appropriate) | Excellent | Very good | Good | Fair | Poor |
|  | Hearing | Self-reported hearing (while using hearing aid if appropriate) | Excellent | Very good | Good | Fair | Poor |
|  | Mobility | Whether respondent has fallen down at all /last year /last 2years | Good outcome | Bad outcome |  |  |  |
|  |  | Whether respondent has fractured hip ever /in last 2 years | Good outcome | Bad outcome |  |  |  |
|  |  | Whether respondent has had joint replacement ever | Good outcome | Bad outcome |  |  |  |
|  |  | Whether respondent has had pain whilst walking | No | Yes |  |  |  |
|  | Cognitive | Today's date: day of month | Good outcome | Bad outcome |  |  |  |
|  |  | Today's date: month | Good outcome | Bad outcome |  |  |  |
|  |  | Today's date: year | Good outcome | Bad outcome |  |  |  |
|  |  | What day of the week it is today? | Good outcome | Bad outcome |  |  |  |
|  |  | Prospective memory test | Good outcome | Bad outcome |  |  |  |
|  |  | Immediate word recall | 1st quartile | 2nd quartile | 3rd quartile | 4th quartile |  |
|  |  | Fluency test (animals) | 1st quantile | 2nd quantile | 3rs quantile | 4th quantile | 5th quartile |
|  |  | Delayed word recall | 1st quantile | 2nd quantile | 3rs quantile | 4th quantile | 5th quartile |

APPENDIX 2

| Genetic | CHR | SNP | BP | A1 | NMISS | BETA | P |
| --- | --- | --- | --- | --- | --- | --- | --- |
| association | 1 | rs653286 | 22971584 | G | 3156 | -0.00139 | 0.637 |
| analysis | 1 | rs291992 | 22977222 | G | 3152 | -0.0009 | 0.7588 |
| results | 1 | rs10914539 | 32715641 | A | 3156 | 0.000198 | 0.9565 |
|  | 1 | rs1004420 | 32729702 | A | 3158 | -0.00242 | 0.5579 |
|  | 1 | rs695161 | 32743866 | G | 3158 | 0.003295 | 0.2587 |
|  | 1 | rs1741981 | 32756439 | G | 3156 | -0.0044 | 0.1667 |
|  | 1 | rs2143749 | 57122467 | G | 3143 | -0.00119 | 0.7022 |
|  | 1 | rs2796516 | 57131323 | A | 3154 | 0.002838 | 0.4827 |
|  | 1 | rs2796509 | 57149336 | A | 3159 | 0.000245 | 0.9344 |
|  | 1 | rs2476599 | 114363459 | A | 3155 | -0.00519 | 0.1142 |
|  | 1 | rs2476601 | 114377568 | A | 3159 | 0.0025 | 0.604 |
|  | 1 | rs12760457 | 114389748 | A | 3152 | 0.005131 | 0.1043 |
|  | 1 | rs1217407 | 114393748 | A | 3159 | -0.00297 | 0.3855 |
|  | 1 | rs11582409 | 114399433 | A | 3160 | 0.003728 | 0.3205 |
|  | 1 | rs1217418 | 114401231 | A | 3157 | -0.00016 | 0.9577 |
|  | 1 | rs1217414 | 114412667 | A | 3158 | -0.00533 | 0.1025 |
|  | 1 | rs17510162 | 114413731 | A | 3157 | 0.00356 | 0.3498 |
|  | 1 | rs952146 | 154368928 | G | 3159 | 0.000269 | 0.9287 |
|  | 1 | rs12083537 | 154381103 | G | 3158 | 0.004694 | 0.1989 |
|  | 1 | rs1386821 | 154382049 | C | 3159 | 0.003652 | 0.3316 |
|  | 1 | rs4075015 | 154389196 | T | 3159 | -0.00141 | 0.6315 |
|  | 1 | rs4601580 | 154394417 | A | 3144 | -0.00378 | 0.2036 |
|  | 1 | rs7549250 | 154404336 | G | 3156 | -0.00372 | 0.2114 |
|  | 1 | rs7518199 | 154407419 | C | 3155 | 0.002973 | 0.3178 |
|  | 1 | rs4453032 | 154414086 | G | 3153 | 0.003542 | 0.2345 |
|  | 1 | rs4537545 | 154418879 | A | 3158 | 0.002644 | 0.3717 |
|  | 1 | rs4129267 | 154426264 | A | 3157 | 0.001864 | 0.5316 |
|  | 1 | rs8192284 | 154426970 | C | 3154 | 0.001908 | 0.5222 |
|  | 1 | rs11265618 | 154430092 | A | 3160 | 0.003289 | 0.3914 |
|  | 1 | rs4240872 | 154436195 | G | 3159 | 0.000664 | 0.8507 |
|  | 1 | rs2229238 | 154437896 | A | 3160 | -0.00036 | 0.9251 |
|  | 1 | rs7526293 | 154444209 | A | 3158 | 0.000339 | 0.9278 |
|  | 1 | rs4845401 | 154941593 | C | 3158 | 0.003132 | 0.2973 |
|  | 1 | rs1800947 | 159683438 | G | 3159 | -0.00344 | 0.5573 |
|  | 1 | rs6030 | 169498975 | G | 3158 | 0.002444 | 0.4354 |
|  | 1 | rs3917854 | 169559035 | A | 3158 | -0.00136 | 0.6687 |
|  | 1 | rs6128 | 169562904 | A | 3157 | 0.006349 | 0.1187 |
|  | 1 | rs6136 | 169563951 | C | 3156 | 9.04E-05 | 0.9842 |
|  | 1 | rs3917811 | 169565393 | A | 3157 | -0.00064 | 0.8855 |
|  | 1 | rs2205896 | 169565565 | A | 3158 | -0.00287 | 0.3447 |
|  | 1 | rs3917792 | 169567832 | A | 3159 | -0.00076 | 0.8328 |
|  | 1 | rs760694 | 169568698 | C | 3158 | 0.001998 | 0.5013 |
|  | 1 | rs2205895 | 169572645 | A | 3142 | 0.002208 | 0.472 |
|  | 1 | rs3917744 | 169577990 | A | 3158 | -0.00038 | 0.9064 |
|  | 1 | rs2076074 | 169578586 | A | 3158 | -0.00679 | 0.05402 |
|  | 1 | rs3917740 | 169579266 | A | 3159 | 0.001578 | 0.6707 |
|  | 1 | rs3917739 | 169579378 | G | 3154 | -0.00431 | 0.1505 |
|  | 1 | rs3917731 | 169580717 | G | 3156 | 0.004399 | 0.174 |
|  | 1 | rs6131 | 169580885 | A | 3158 | -0.01013 | **0.009649** |
|  | 1 | rs3917729 | 169581130 | A | 3156 | -0.01038 | *0.02292* |
|  | 1 | rs3917727 | 169581258 | G | 3158 | 0.002961 | 0.3391 |
|  | 1 | rs2236868 | 169583996 | A | 3157 | -0.00138 | 0.6416 |
|  | 1 | rs2244529 | 169587032 | G | 3153 | -0.00838 | *0.01048* |
|  | 1 | rs3917688 | 169591080 | G | 3158 | 0.000622 | 0.832 |
|  | 1 | rs3917682 | 169591548 | C | 3145 | 0.000294 | 0.9219 |
|  | 1 | rs2236866 | 169596313 | A | 3159 | 0.002136 | 0.4709 |
|  | 1 | rs1076637 | 169697444 | A | 3160 | -0.00089 | 0.8395 |
|  | 1 | rs2076059 | 169698921 | A | 3156 | -0.00127 | 0.6667 |
|  | 1 | rs3917412 | 169700502 | A | 3159 | 0.000558 | 0.8608 |
|  | 1 | rs6700734 | 172630811 | G | 3157 | 0.004448 | 0.1781 |
|  | 1 | rs929087 | 172632057 | A | 3159 | 0.002484 | 0.3969 |
|  | 1 | rs800292 | 196642233 | A | 3157 | 7.40E-05 | 0.9831 |
|  | 1 | rs4390174 | 206932451 | G | 3158 | 0.002084 | 0.4998 |
|  | 1 | rs6673928 | 206937245 | A | 2985 | 0.001026 | 0.7648 |
|  | 1 | rs3024498 | 206941529 | G | 3160 | 0.000799 | 0.8051 |
|  | 1 | rs3024496 | 206941864 | A | 3152 | -0.00302 | 0.3037 |
|  | 1 | rs3024495 | 206942413 | A | 3158 | 0.001817 | 0.6556 |
|  | 1 | rs3024493 | 206943968 | A | 3160 | 0.001756 | 0.6664 |
|  | 1 | rs1554286 | 206944233 | A | 3158 | -0.00333 | 0.3897 |
|  | 1 | rs1518111 | 206944645 | A | 3160 | -0.0037 | 0.3111 |
|  | 1 | rs1800871 | 206946634 | A | 3158 | -0.00327 | 0.3531 |
|  | 1 | rs1800896 | 206946897 | A | 3150 | -0.00325 | 0.2693 |
|  | 1 | rs1800890 | 206949365 | A | 3158 | 0.001852 | 0.5344 |
|  | 1 | rs17015767 | 206951398 | C | 3159 | 0.001868 | 0.5736 |
|  | 1 | rs10494879 | 206952204 | C | 3159 | 0.001294 | 0.6624 |
|  | 1 | rs6686931 | 206955651 | A | 3159 | -0.00223 | 0.5426 |
|  | 1 | rs6667202 | 206957092 | C | 3159 | 0.001815 | 0.5373 |
|  | 1 | rs17015865 | 206958587 | A | 3156 | 0.00131 | 0.6945 |
|  | 1 | rs12086634 | 209880259 | C | 3160 | -0.00355 | 0.3194 |
|  | 1 | rs11119328 | 209885318 | A | 3159 | -0.00365 | 0.3357 |
|  | 1 | rs6672256 | 209894043 | T | 3160 | -0.00393 | 0.268 |
|  | 1 | rs932335 | 209905734 | G | 3158 | -0.00339 | 0.3383 |
|  | 1 | rs1805087 | 237048500 | G | 3158 | 0.004973 | 0.181 |
|  | 2 | rs1042031 | 21225753 | A | 3159 | 0.000741 | 0.8445 |
|  | 2 | rs2678379 | 21226560 | A | 3159 | 0.003279 | 0.3668 |
|  | 2 | rs1801701 | 21228827 | A | 3159 | -0.00646 | 0.1884 |
|  | 2 | rs693 | 21232195 | G | 3151 | 0.005368 | 0.06624 |
|  | 2 | rs11126598 | 21240364 | A | 3157 | 0.00069 | 0.8313 |
|  | 2 | rs10199768 | 21244000 | A | 3159 | -0.00277 | 0.3453 |
|  | 2 | rs11676704 | 21244358 | C | 3160 | -0.00176 | 0.6566 |
|  | 2 | rs520354 | 21259612 | G | 3144 | 6.79E-06 | 0.9982 |
|  | 2 | rs531819 | 21263639 | A | 3160 | 0.002773 | 0.4945 |
|  | 2 | rs1367117 | 21263900 | A | 3158 | -0.00138 | 0.6584 |
|  | 2 | rs12469600 | 113572357 | G | 3159 | -7.55E-05 | 0.9817 |
|  | 2 | rs3917368 | 113582782 | A | 3153 | 0.000591 | 0.8462 |
|  | 2 | rs1143643 | 113588302 | A | 3159 | 0.000597 | 0.8443 |
|  | 2 | rs1143634 | 113590390 | A | 3157 | -0.00385 | 0.2586 |
|  | 2 | rs16944 | 113594867 | A | 3160 | 0.00284 | 0.3631 |
|  | 2 | rs4848306 | 113598107 | A | 3142 | -0.00193 | 0.5136 |
|  | 2 | rs10169916 | 113603728 | A | 3159 | 0.002862 | 0.3594 |
|  | 2 | rs315928 | 113868263 | A | 3159 | -0.00378 | 0.3269 |
|  | 2 | rs1688072 | 113869347 | G | 3143 | -0.00335 | 0.3851 |
|  | 2 | rs315931 | 113869843 | C | 3158 | 0.000327 | 0.9192 |
|  | 2 | rs4251961 | 113874467 | G | 3160 | 0.001591 | 0.5993 |
|  | 2 | rs2637988 | 113876779 | G | 3158 | 0.0012 | 0.6929 |
|  | 2 | rs4251985 | 113877413 | A | 3159 | 0.001699 | 0.6105 |
|  | 2 | rs315936 | 113880947 | A | 3157 | 0.001303 | 0.6981 |
|  | 2 | rs1794066 | 113886350 | G | 3160 | 0.001111 | 0.7139 |
|  | 2 | rs3087266 | 113889100 | A | 3158 | -0.00036 | 0.9317 |
|  | 2 | rs579543 | 113889631 | A | 3160 | 0.000606 | 0.8544 |
|  | 2 | rs315952 | 113890304 | G | 3155 | -0.00185 | 0.5723 |
|  | 2 | rs396201 | 113891775 | G | 3157 | 0.000617 | 0.8478 |
|  | 2 | rs397211 | 113892141 | G | 3155 | 0.000479 | 0.8816 |
|  | 2 | rs315949 | 113892774 | A | 3159 | 0.001346 | 0.6556 |
|  | 2 | rs315946 | 113893864 | A | 3160 | -0.0042 | 0.328 |
|  | 2 | rs315943 | 113894338 | G | 3160 | 0.001726 | 0.5672 |
|  | 2 | rs2902452 | 113895516 | A | 3156 | 0.000771 | 0.7996 |
|  | 2 | rs1374281 | 113898789 | C | 3157 | -3.04E-05 | 0.992 |
|  | 2 | rs895496 | 113899025 | A | 3150 | 0.000217 | 0.9428 |
|  | 2 | rs315958 | 113900851 | A | 3159 | -0.0015 | 0.6393 |
|  | 2 | rs315957 | 113901313 | A | 3154 | -0.00168 | 0.5999 |
|  | 2 | rs2111485 | 163110536 | A | 3157 | 0.00418 | 0.1609 |
|  | 2 | rs1990760 | 163124051 | G | 3155 | 0.004398 | 0.1403 |
|  | 2 | rs3087243 | 204738919 | A | 3158 | 0.000114 | 0.9691 |
|  | 2 | rs2290708 | 219252377 | A | 3156 | 0.001257 | 0.7061 |
|  | 2 | rs3816560 | 219254842 | G | 3152 | 0.002042 | 0.5376 |
|  | 2 | rs2279015 | 219259270 | A | 3154 | 0.000764 | 0.8002 |
|  | 2 | rs17208239 | 227597697 | A | 3159 | -0.00068 | 0.9019 |
|  | 2 | rs17208470 | 227603905 | A | 3157 | 0.001924 | 0.6896 |
|  | 2 | rs6725330 | 227666857 | G | 3155 | -0.00653 | 0.151 |
|  | 3 | rs1801282 | 12393125 | G | 3160 | 0.004517 | 0.3234 |
|  | 3 | rs2881654 | 12396955 | A | 3157 | 0.005993 | 0.1843 |
|  | 3 | rs3856806 | 12475557 | A | 3159 | 0.003753 | 0.4 |
|  | 3 | rs9790292 | 30661210 | A | 3141 | -0.00103 | 0.7283 |
|  | 3 | rs5020833 | 30670425 | G | 3160 | 0.003776 | 0.2331 |
|  | 3 | rs6809777 | 30672362 | A | 3159 | 0.00096 | 0.7696 |
|  | 3 | rs13083158 | 30675957 | C | 3159 | 0.001513 | 0.6246 |
|  | 3 | rs11924422 | 30677484 | C | 3156 | -0.00277 | 0.3466 |
|  | 3 | rs17025857 | 30681095 | G | 3158 | 0.002942 | 0.3602 |
|  | 3 | rs3773627 | 30694413 | A | 3158 | -0.00534 | 0.235 |
|  | 3 | rs352143 | 52264907 | G | 3160 | -0.0007 | 0.8451 |
|  | 3 | rs11717574 | 52268246 | G | 3157 | 0.001215 | 0.7602 |
|  | 3 | rs353547 | 52268866 | A | 3159 | -0.00523 | 0.08492 |
|  | 3 | rs4082828 | 52274742 | C | 3156 | 0.003624 | 0.4123 |
|  | 3 | rs2886666 | 159695933 | A | 3156 | -0.0092 | *0.01446* |
|  | 3 | rs9811792 | 159696998 | G | 3159 | 6.23E-05 | 0.9833 |
|  | 3 | rs7372767 | 159698866 | G | 3156 | -0.00014 | 0.963 |
|  | 3 | rs747825 | 159699519 | A | 3160 | -0.00866 | *0.02159* |
|  | 3 | rs755004 | 159700325 | A | 3154 | -0.00049 | 0.9043 |
|  | 3 | rs662959 | 159701231 | A | 3159 | 3.11E-05 | 0.9942 |
|  | 3 | rs2243123 | 159709651 | G | 3156 | 0.004715 | 0.143 |
|  | 3 | rs583911 | 159710390 | G | 3160 | -0.00475 | 0.1089 |
|  | 3 | rs640039 | 159713846 | G | 3160 | 0.000753 | 0.8599 |
|  | 3 | rs2243143 | 159714802 | A | 3158 | 0.005511 | 0.06384 |
|  | 3 | rs2243148 | 159715411 | G | 3160 | 0.004647 | 0.1492 |
|  | 3 | rs668998 | 159715551 | G | 3160 | -0.00504 | 0.08903 |
|  | 3 | rs2243154 | 159716242 | A | 3159 | -0.00749 | 0.1429 |
|  | 3 | rs485497 | 159719132 | A | 3153 | -0.00345 | 0.2428 |
|  | 3 | rs9852519 | 159720628 | A | 3155 | -0.00813 | **0.007704** |
|  | 3 | rs598638 | 159720817 | A | 3158 | 0.000227 | 0.9549 |
|  | 3 | rs4679868 | 159724154 | A | 3155 | -0.00827 | **0.006196** |
|  | 3 | rs182052 | 186560782 | A | 3155 | -0.00787 | *0.01095* |
|  | 3 | rs822391 | 186563803 | G | 3159 | -0.00608 | 0.09703 |
|  | 3 | rs822396 | 186566877 | G | 3157 | -0.00724 | 0.05262 |
|  | 3 | rs12495941 | 186568180 | A | 3156 | 0.005248 | 0.09245 |
|  | 3 | rs7649121 | 186568785 | T | 3158 | -0.00262 | 0.4923 |
|  | 3 | rs2241766 | 186570892 | C | 3154 | 0.008412 | 0.05673 |
|  | 3 | rs1501299 | 186571123 | A | 3160 | 0.002927 | 0.3801 |
|  | 3 | rs3821799 | 186571486 | A | 3158 | 0.003616 | 0.2218 |
|  | 3 | rs3774262 | 186571814 | A | 3157 | 0.008538 | 0.0564 |
|  | 3 | rs6773957 | 186573705 | A | 3157 | 0.006129 | *0.04256* |
|  | 3 | rs1063537 | 186574075 | A | 3158 | 0.008718 | 0.05102 |
|  | 4 | rs3775779 | 70709207 | T | 3159 | 0.003652 | 0.2514 |
|  | 4 | rs1220704 | 70711528 | A | 3157 | 0.002746 | 0.4972 |
|  | 4 | rs4149535 | 70714171 | C | 3157 | 0.002731 | 0.4652 |
|  | 4 | rs1220715 | 70716627 | A | 3158 | -0.00611 | 0.1734 |
|  | 4 | rs4149533 | 70718036 | G | 3157 | 3.22E-05 | 0.9919 |
|  | 4 | rs3822173 | 70723815 | A | 3160 | -0.00319 | 0.5897 |
|  | 4 | rs3775770 | 70724270 | A | 3158 | -0.00091 | 0.7801 |
|  | 4 | rs1881668 | 70725456 | G | 3159 | -0.00091 | 0.7829 |
|  | 4 | rs16849928 | 74599710 | G | 3160 | 0.000785 | 0.7923 |
|  | 4 | rs4694637 | 74612834 | G | 3154 | -0.00054 | 0.857 |
|  | 4 | rs17202249 | 74617243 | A | 3159 | -0.00568 | 0.2473 |
|  | 4 | rs12508866 | 142565693 | G | 3159 | 0.003692 | 0.2903 |
|  | 4 | rs17461269 | 142573348 | T | 3157 | -0.00321 | 0.2946 |
|  | 4 | rs1519552 | 142576023 | A | 3158 | 0.001303 | 0.6916 |
|  | 4 | rs13139573 | 142616194 | A | 3155 | -0.00037 | 0.899 |
|  | 4 | rs7349640 | 142650901 | G | 3160 | -0.00131 | 0.7423 |
|  | 4 | rs1800791 | 155483309 | A | 3158 | -0.00394 | 0.3415 |
|  | 4 | rs1800790 | 155483708 | A | 3158 | 0.002477 | 0.511 |
|  | 4 | rs2070011 | 155511897 | A | 3157 | 0.003018 | 0.3197 |
|  | 5 | rs10940495 | 55262660 | G | 3158 | 0.002735 | 0.4023 |
|  | 5 | rs6450358 | 55270004 | G | 3153 | 0.01147 | *0.01253* |
|  | 5 | rs10434696 | 158698668 | A | 3140 | -0.00321 | 0.306 |
|  | 5 | rs7730126 | 158729947 | A | 3158 | -0.00191 | 0.5543 |
|  | 5 | rs1549922 | 158731548 | A | 3160 | 0.000108 | 0.971 |
|  | 5 | rs10072923 | 158735776 | G | 3159 | 0.002695 | 0.4729 |
|  | 5 | rs1865014 | 158739088 | A | 3160 | 0.003887 | 0.2905 |
|  | 5 | rs3181226 | 158740530 | C | 3159 | -0.00021 | 0.9566 |
|  | 5 | rs3212227 | 158742950 | C | 3158 | 0.002847 | 0.4505 |
|  | 5 | rs2853696 | 158744660 | A | 3152 | 0.003347 | 0.363 |
|  | 5 | rs2853694 | 158749088 | A | 3154 | -0.00029 | 0.9229 |
|  | 5 | rs2569253 | 158750993 | A | 3158 | 0.001867 | 0.5207 |
|  | 5 | rs2569254 | 158751249 | A | 3160 | -1.07E-05 | 0.9977 |
|  | 5 | rs1003199 | 158755566 | A | 3159 | -0.00155 | 0.5937 |
|  | 5 | rs1433048 | 158755845 | G | 3152 | 0.000873 | 0.8134 |
|  | 5 | rs2546893 | 158755960 | A | 3160 | -0.0003 | 0.9191 |
|  | 5 | rs6894567 | 158756968 | G | 3159 | 0.004882 | 0.1967 |
|  | 5 | rs2546890 | 158759900 | G | 3159 | -0.00172 | 0.5579 |
|  | 5 | rs10052709 | 158760477 | C | 3159 | -0.00337 | 0.414 |
|  | 5 | rs7709212 | 158764177 | G | 3159 | 0.000111 | 0.9719 |
|  | 5 | rs6868898 | 158764420 | G | 3156 | -0.0006 | 0.8479 |
|  | 6 | rs5985 | 6318795 | A | 3129 | -0.00543 | 0.1014 |
|  | 6 | rs2844484 | 31536224 | A | 3159 | -0.00018 | 0.953 |
|  | 6 | rs2844482 | 31539767 | A | 3158 | -0.00096 | 0.8139 |
|  | 6 | rs1041981 | 31540784 | A | 3153 | 0.000914 | 0.7648 |
|  | 6 | rs1800629 | 31543031 | A | 3151 | 0.004707 | 0.2011 |
|  | 6 | rs1800610 | 31543827 | A | 3158 | -0.00126 | 0.8179 |
|  | 6 | rs2256965 | 31555130 | A | 3159 | -0.00249 | 0.4024 |
|  | 6 | rs2256974 | 31555392 | A | 3158 | -0.00397 | 0.302 |
|  | 6 | rs2016520 | 35378778 | G | 3159 | 0.000901 | 0.8056 |
|  | 7 | rs1476483 | 22731199 | G | 3156 | 0.001967 | 0.5905 |
|  | 7 | rs6952003 | 22752705 | A | 3157 | -0.0005 | 0.8803 |
|  | 7 | rs7805828 | 22758562 | A | 3158 | 0.00298 | 0.3179 |
|  | 7 | rs1880241 | 22759469 | G | 3152 | 0.001613 | 0.5821 |
|  | 7 | rs4719714 | 22760713 | T | 3160 | 0.001329 | 0.7009 |
|  | 7 | rs2056576 | 22761202 | A | 3159 | 0.001222 | 0.7001 |
|  | 7 | rs12700386 | 22763009 | C | 3156 | 0.002611 | 0.4806 |
|  | 7 | rs1800795 | 22766645 | G | 3158 | -0.004 | 0.175 |
|  | 7 | rs2069832 | 22767433 | A | 3156 | -0.00337 | 0.2541 |
|  | 7 | rs2069833 | 22767664 | G | 3155 | -0.0036 | 0.2232 |
|  | 7 | rs2069840 | 22768572 | C | 3160 | 0.000741 | 0.8101 |
|  | 7 | rs10242595 | 22774231 | A | 3157 | 0.002742 | 0.396 |
|  | 7 | rs11766273 | 22775663 | A | 3159 | 0.00772 | 0.1805 |
|  | 7 | rs4724445 | 45923200 | A | 3157 | 0.001917 | 0.6133 |
|  | 7 | rs3763497 | 45925348 | A | 3159 | 0.001513 | 0.6279 |
|  | 7 | rs4619 | 45932669 | G | 3157 | 0.000654 | 0.8304 |
|  | 7 | rs9658239 | 45934159 | G | 3160 | -6.52E-05 | 0.9879 |
|  | 7 | rs12702181 | 45945469 | G | 3160 | 0.002133 | 0.4918 |
|  | 7 | rs6670 | 45952254 | T | 3158 | -0.00125 | 0.7194 |
|  | 7 | rs2453839 | 45953573 | G | 3160 | -0.00291 | 0.4309 |
|  | 7 | rs3110697 | 45955029 | A | 3136 | 0.001561 | 0.601 |
|  | 7 | rs2471551 | 45957055 | C | 3160 | -0.00128 | 0.7294 |
|  | 7 | rs2132572 | 45961545 | A | 3160 | 0.004629 | 0.1845 |
|  | 7 | rs2269829 | 94936129 | G | 3157 | 0.004348 | 0.1791 |
|  | 7 | rs662 | 94937446 | G | 3158 | 0.003491 | 0.2772 |
|  | 7 | rs854560 | 94946084 | T | 3159 | 0.003263 | 0.2833 |
|  | 7 | rs7785846 | 95033841 | A | 3159 | 0.001261 | 0.7127 |
|  | 7 | rs6950982 | 100766603 | G | 3155 | -0.00116 | 0.745 |
|  | 7 | rs2227631 | 100769538 | G | 3160 | 0.000776 | 0.7984 |
|  | 7 | rs2227672 | 100775686 | A | 3146 | 0.001669 | 0.7119 |
|  | 7 | rs11178 | 100781084 | G | 3159 | -0.00373 | 0.2079 |
|  | 7 | rs1050813 | 100781615 | A | 3159 | 0.003986 | 0.2537 |
|  | 7 | rs1050955 | 100782460 | A | 3155 | -0.00141 | 0.705 |
|  | 7 | rs13238709 | 100783736 | A | 3157 | -0.00389 | 0.1888 |
|  | 7 | rs2004640 | 128578301 | C | 3118 | 0.003712 | 0.21 |
|  | 7 | rs7808907 | 128584084 | G | 3158 | -0.00213 | 0.4711 |
|  | 7 | rs13242262 | 128591364 | T | 3160 | 0.000642 | 0.8308 |
|  | 7 | rs10488630 | 128593948 | G | 3158 | -0.00158 | 0.6055 |
|  | 8 | rs1534649 | 19799641 | A | 3156 | -0.00305 | 0.2997 |
|  | 8 | rs13266204 | 19800005 | G | 3156 | 0.00242 | 0.5073 |
|  | 8 | rs3779788 | 19803093 | A | 3151 | -0.00373 | 0.3601 |
|  | 8 | rs253 | 19811417 | A | 3157 | -0.00125 | 0.6652 |
|  | 8 | rs256 | 19811967 | A | 3160 | 0.000397 | 0.9244 |
|  | 8 | rs270 | 19813676 | A | 3158 | 0.002265 | 0.5703 |
|  | 8 | rs281 | 19815023 | T | 3159 | 0.002542 | 0.4373 |
|  | 8 | rs283 | 19815098 | A | 3157 | 0.000176 | 0.9617 |
|  | 8 | rs285 | 19815189 | A | 3156 | -0.00131 | 0.6533 |
|  | 8 | rs291 | 19815852 | G | 3157 | -0.00201 | 0.5579 |
|  | 8 | rs301 | 19816934 | G | 3157 | -0.00203 | 0.5534 |
|  | 8 | rs325 | 19819328 | G | 3156 | -0.00436 | 0.3588 |
|  | 8 | rs327 | 19819536 | C | 3160 | -0.0037 | 0.2574 |
|  | 8 | rs328 | 19819724 | G | 3159 | -0.00448 | 0.3445 |
|  | 8 | rs10099160 | 19821815 | C | 3156 | -0.00102 | 0.7609 |
|  | 8 | rs11570892 | 19823617 | G | 3159 | -0.00047 | 0.9077 |
|  | 8 | rs13269094 | 30896151 | C | 3160 | -0.00325 | 0.4246 |
|  | 8 | rs10808311 | 30898262 | G | 3159 | -0.00254 | 0.3921 |
|  | 8 | rs11574218 | 30937790 | A | 3160 | -0.00156 | 0.6073 |
|  | 8 | rs11574227 | 30940392 | A | 3156 | 0.001254 | 0.8343 |
|  | 8 | rs11574276 | 30959000 | T | 3159 | 0.003854 | 0.3177 |
|  | 8 | rs4733224 | 30967369 | G | 3152 | -0.00035 | 0.9091 |
|  | 8 | rs2725351 | 30977446 | G | 3159 | 0.001114 | 0.7059 |
|  | 8 | rs2725361 | 30997326 | G | 3157 | 0.00093 | 0.7543 |
|  | 8 | rs10954780 | 31021717 | C | 3147 | 0.002107 | 0.4744 |
|  | 8 | rs1346044 | 31024654 | G | 3159 | -0.00142 | 0.6695 |
|  | 8 | rs10954781 | 31029925 | G | 3144 | -8.58E-05 | 0.979 |
|  | 8 | rs4994 | 37823798 | G | 3158 | 0.003169 | 0.5685 |
|  | 8 | rs3747811 | 42129505 | A | 3158 | -0.00043 | 0.884 |
|  | 8 | rs5029748 | 42140549 | A | 3158 | -0.00029 | 0.9292 |
|  | 8 | rs10958713 | 42180716 | A | 3159 | 0.002817 | 0.36 |
|  | 8 | rs1799998 | 143999600 | G | 3160 | 0.004604 | 0.1194 |
|  | 9 | rs1927911 | 120470054 | A | 3158 | 0.001994 | 0.5491 |
|  | 9 | rs1927907 | 120472764 | A | 3155 | -0.0031 | 0.4672 |
|  | 9 | rs5030717 | 120473834 | G | 3143 | -0.00388 | 0.4158 |
|  | 9 | rs5030728 | 120474282 | A | 3159 | 0.00283 | 0.3743 |
|  | 10 | rs11101318 | 49625583 | A | 3160 | -0.00135 | 0.6467 |
|  | 10 | rs10508901 | 49627398 | A | 3156 | -0.00157 | 0.6075 |
|  | 10 | rs12358297 | 49640051 | C | 3159 | 0.001401 | 0.6822 |
|  | 10 | rs3740286 | 90751340 | G | 3160 | 0.003128 | 0.3121 |
|  | 10 | rs10509561 | 90751912 | A | 3160 | -0.00497 | 0.1017 |
|  | 10 | rs7097467 | 90753244 | G | 3160 | 0.004061 | 0.3799 |
|  | 10 | rs1926195 | 90753758 | G | 3153 | 0.003814 | 0.1915 |
|  | 10 | rs1571011 | 90757787 | C | 3159 | 0.00258 | 0.3931 |
|  | 10 | rs2147420 | 90759613 | A | 3160 | -0.00259 | 0.3849 |
|  | 10 | rs9658727 | 90761865 | G | 3159 | 0.000267 | 0.948 |
|  | 10 | rs2031613 | 90766924 | G | 3156 | -0.00302 | 0.3472 |
|  | 10 | rs7911226 | 90768965 | G | 3155 | 0.000284 | 0.9302 |
|  | 10 | rs9658761 | 90769886 | A | 3160 | 0.002751 | 0.5645 |
|  | 10 | rs1051070 | 90774772 | T | 3159 | -0.00623 | 0.1695 |
|  | 10 | rs4919686 | 104592249 | C | 3158 | 0.00116 | 0.7214 |
|  | 10 | rs1801253 | 115805056 | C | 3088 | -0.00305 | 0.3629 |
|  | 11 | rs5896 | 46745003 | A | 3153 | 0.00046 | 0.9118 |
|  | 11 | rs11039482 | 48009074 | A | 3159 | 0.004049 | 0.3287 |
|  | 11 | rs7130876 | 48050995 | G | 3158 | 0.001664 | 0.6451 |
|  | 11 | rs6485802 | 48051442 | C | 3158 | -0.00144 | 0.6562 |
|  | 11 | rs7934659 | 48066374 | A | 3160 | 0.000578 | 0.8607 |
|  | 11 | rs11601310 | 48085189 | A | 3150 | 0.005604 | 0.1209 |
|  | 11 | rs10734561 | 48097480 | G | 3156 | 0.00072 | 0.8274 |
|  | 11 | rs1039484 | 48097948 | A | 3155 | 0.003018 | 0.3406 |
|  | 11 | rs7122335 | 48106486 | G | 3159 | 0.001614 | 0.6504 |
|  | 11 | rs1910364 | 48113232 | A | 3158 | 0.001037 | 0.7699 |
|  | 11 | rs7130402 | 48115606 | G | 3159 | 0.00037 | 0.9462 |
|  | 11 | rs4356181 | 48132197 | A | 3153 | -0.00559 | 0.1768 |
|  | 11 | rs2270993 | 48145166 | A | 3148 | 0.001524 | 0.7163 |
|  | 11 | rs10769317 | 48150712 | A | 3159 | 0.003104 | 0.3561 |
|  | 11 | rs1566732 | 48151494 | C | 3159 | 1.20E-05 | 0.9968 |
|  | 11 | rs2047812 | 48162042 | A | 3157 | 0.004775 | 0.2005 |
|  | 11 | rs7124275 | 48162305 | G | 3156 | 0.001036 | 0.7348 |
|  | 11 | rs1566729 | 48164721 | A | 3155 | 0.003795 | 0.3335 |
|  | 11 | rs1566728 | 48164847 | G | 3154 | 0.00356 | 0.3641 |
|  | 11 | rs1784223 | 65380248 | G | 3160 | 0.001715 | 0.5768 |
|  | 11 | rs7116336 | 100962873 | A | 3158 | 0.00069 | 0.8896 |
|  | 11 | rs4987876 | 108092637 | A | 3156 | -0.0049 | 0.3378 |
|  | 11 | rs627418 | 108131225 | A | 3159 | 0.003444 | 0.2457 |
|  | 11 | rs3092991 | 108140516 | G | 3160 | -0.003 | 0.4732 |
|  | 11 | rs228593 | 108141134 | A | 3159 | 0.000816 | 0.7921 |
|  | 11 | rs611646 | 108177097 | T | 3154 | -0.00085 | 0.774 |
|  | 11 | rs373759 | 108220657 | A | 3159 | 0.00085 | 0.7837 |
|  | 11 | rs3882891 | 112014761 | C | 3159 | -0.00052 | 0.8616 |
|  | 11 | rs549908 | 112020916 | C | 3158 | 0.005637 | 0.07592 |
|  | 11 | rs5744256 | 112022848 | G | 3158 | -0.00254 | 0.4467 |
|  | 11 | rs1834481 | 112023827 | C | 3157 | -0.00249 | 0.4545 |
|  | 11 | rs360722 | 112026703 | A | 3155 | -0.01498 | **0.002051** |
|  | 11 | rs4937113 | 112029721 | A | 3158 | -0.0007 | 0.8143 |
|  | 11 | rs795467 | 112031080 | A | 3159 | 0.006267 | 0.05512 |
|  | 11 | rs2043055 | 112031624 | G | 3158 | 0.002109 | 0.4821 |
|  | 11 | rs1946519 | 112035507 | A | 3154 | -0.00171 | 0.5694 |
|  | 11 | rs1293344 | 112037384 | G | 3160 | 0.00628 | 0.05545 |
|  | 11 | rs11214105 | 112037653 | A | 3155 | 0.006667 | *0.04187* |
|  | 11 | rs618923 | 116654159 | G | 3160 | 0.004033 | 0.2427 |
|  | 11 | rs603446 | 116654435 | A | 3159 | 0.000205 | 0.9444 |
|  | 11 | rs619054 | 116660813 | A | 3137 | 0.003301 | 0.3406 |
|  | 11 | rs662799 | 116663707 | G | 3157 | 0.00821 | 0.2112 |
|  | 11 | rs1729410 | 116665661 | G | 3149 | 0.000646 | 0.8286 |
|  | 11 | rs6589568 | 116670738 | G | 3141 | 0.0052 | 0.1505 |
|  | 11 | rs1263167 | 116677723 | G | 3157 | -0.00123 | 0.7437 |
|  | 11 | rs5110 | 116691634 | A | 3160 | 0.009454 | 0.07081 |
|  | 11 | rs888245 | 116723737 | C | 3156 | -0.00616 | 0.1338 |
|  | 11 | rs888246 | 116724232 | A | 3133 | -0.00506 | 0.2856 |
|  | 11 | rs625145 | 116727936 | A | 3158 | -0.00882 | *0.01788* |
|  | 11 | rs11216164 | 116734545 | A | 3159 | 0.002091 | 0.5007 |
|  | 11 | rs573549 | 116736679 | A | 3156 | -5.28E-05 | 0.9862 |
|  | 11 | rs548638 | 116737093 | C | 3157 | -0.00206 | 0.5629 |
|  | 12 | rs11172113 | 57527283 | G | 3157 | 0.001102 | 0.7118 |
|  | 12 | rs4759044 | 57530670 | G | 3159 | -0.00217 | 0.4545 |
|  | 12 | rs1385526 | 57532749 | C | 3160 | -0.00115 | 0.7038 |
|  | 12 | rs715948 | 57532982 | A | 3160 | -0.00099 | 0.7492 |
|  | 12 | rs1799986 | 57535266 | A | 3151 | 0.011 | **0.006519** |
|  | 12 | rs7968719 | 57540751 | C | 3153 | -0.00361 | 0.2136 |
|  | 12 | rs7398375 | 57540848 | G | 3122 | -0.00025 | 0.9383 |
|  | 12 | rs10876966 | 57543572 | A | 3159 | -0.0011 | 0.755 |
|  | 12 | rs1800168 | 57592557 | G | 3160 | -0.00265 | 0.3983 |
|  | 12 | rs1800159 | 57593894 | A | 3148 | -0.00087 | 0.7832 |
|  | 12 | rs7956957 | 57602815 | G | 3120 | -0.00203 | 0.5148 |
|  | 12 | rs2193050 | 68547193 | A | 3159 | -0.00063 | 0.8439 |
|  | 12 | rs2069727 | 68548223 | G | 3158 | -0.00125 | 0.6674 |
|  | 12 | rs2069718 | 68550162 | A | 3155 | -0.00026 | 0.9298 |
|  | 12 | rs6219 | 102790192 | A | 3159 | -0.00136 | 0.786 |
|  | 12 | rs6214 | 102793569 | A | 3155 | 0.005925 | *0.04626* |
|  | 12 | rs1520220 | 102796522 | G | 3160 | -0.00098 | 0.801 |
|  | 12 | rs5742694 | 102799236 | C | 3157 | 0.002738 | 0.4249 |
|  | 12 | rs17727841 | 102809630 | G | 3158 | 0.0036 | 0.3589 |
|  | 12 | rs5742678 | 102814332 | G | 3156 | 0.002387 | 0.4823 |
|  | 12 | rs972936 | 102824921 | A | 3159 | 0.002533 | 0.4555 |
|  | 12 | rs9989002 | 102850223 | A | 3160 | 0.002713 | 0.4111 |
|  | 12 | rs5742632 | 102856474 | G | 3159 | 0.001183 | 0.7387 |
|  | 12 | rs5742629 | 102857263 | G | 3157 | 0.001251 | 0.7115 |
|  | 12 | rs10778176 | 102862979 | A | 3158 | 0.000864 | 0.7988 |
|  | 12 | rs1019731 | 102864425 | A | 3160 | -0.00013 | 0.9745 |
|  | 12 | rs12821878 | 102867667 | A | 3151 | -0.00096 | 0.7776 |
|  | 12 | rs2162679 | 102871259 | G | 3158 | -0.00121 | 0.7649 |
|  | 13 | rs17222814 | 31299553 | A | 3158 | -0.00282 | 0.5604 |
|  | 13 | rs6046 | 113773159 | A | 3156 | 0.000486 | 0.9187 |
|  | 14 | rs2301106 | 62166563 | G | 3159 | -0.00478 | 0.3017 |
|  | 14 | rs1951795 | 62171426 | A | 3156 | 0.000659 | 0.8662 |
|  | 14 | rs11158358 | 62198954 | C | 3158 | -0.00279 | 0.4949 |
|  | 15 | rs6493487 | 51513729 | G | 3157 | 0.001053 | 0.7537 |
|  | 15 | rs2899472 | 51516055 | A | 3156 | 0.002229 | 0.5084 |
|  | 15 | rs12439137 | 51516304 | G | 3157 | 0.005115 | 0.2567 |
|  | 15 | rs8023263 | 51517597 | C | 3158 | 0.001203 | 0.6851 |
|  | 15 | rs2414095 | 51524292 | A | 3099 | 0.000971 | 0.7588 |
|  | 15 | rs10519295 | 51532647 | G | 3160 | 0.003246 | 0.5276 |
|  | 15 | rs4775936 | 51536022 | A | 3160 | -0.00051 | 0.8617 |
|  | 15 | rs10459592 | 51536141 | A | 3159 | -0.00014 | 0.9629 |
|  | 15 | rs12591359 | 51539368 | A | 3149 | -0.00022 | 0.9415 |
|  | 15 | rs12911554 | 51542757 | G | 3159 | 0.00203 | 0.4917 |
|  | 15 | rs7172156 | 51546298 | A | 3160 | 0.00216 | 0.4657 |
|  | 15 | rs2414099 | 51548782 | G | 3159 | 0.000246 | 0.9513 |
|  | 15 | rs4545755 | 51549044 | A | 3157 | -0.00109 | 0.7124 |
|  | 15 | rs2305707 | 51569410 | G | 3158 | 0.000798 | 0.8504 |
|  | 15 | rs2470155 | 51590753 | A | 3157 | -0.00032 | 0.9481 |
|  | 15 | rs17523880 | 51592543 | A | 3158 | 0.000555 | 0.8921 |
|  | 15 | rs2470152 | 51594972 | A | 3160 | 0.001033 | 0.7241 |
|  | 15 | rs3751592 | 51606578 | G | 3131 | -0.00052 | 0.87 |
|  | 15 | rs3751591 | 51606710 | G | 3160 | 0.003791 | 0.3372 |
|  | 15 | rs1902584 | 51611654 | A | 3157 | 0.000152 | 0.977 |
|  | 15 | rs1004982 | 51613811 | G | 3157 | 0.001058 | 0.7255 |
|  | 15 | rs4774585 | 51616480 | A | 3158 | -0.00088 | 0.802 |
|  | 15 | rs2445762 | 51617708 | G | 3155 | -0.00284 | 0.3816 |
|  | 15 | rs2470144 | 51621725 | A | 3157 | 0.002252 | 0.4417 |
|  | 15 | rs7174997 | 51622128 | A | 3155 | 0.001228 | 0.7497 |
|  | 15 | rs1961177 | 51625078 | A | 3155 | 0.005056 | 0.2564 |
|  | 15 | rs1077834 | 58723479 | G | 3159 | -0.00336 | 0.336 |
|  | 15 | rs1800588 | 58723675 | A | 3160 | -0.0025 | 0.4751 |
|  | 15 | rs2070895 | 58723939 | A | 3160 | -0.00326 | 0.3517 |
|  | 15 | rs2470893 | 75019449 | A | 3159 | -0.00013 | 0.9657 |
|  | 15 | rs11247380 | 99440731 | A | 3156 | -3.16E-05 | 0.9921 |
|  | 15 | rs3743259 | 99443161 | G | 3157 | -0.00024 | 0.9421 |
|  | 15 | rs2293117 | 99478713 | G | 3157 | 0.003194 | 0.2777 |
|  | 16 | rs1864163 | 56997233 | A | 3159 | 0.004661 | 0.1713 |
|  | 16 | rs9929488 | 56998572 | C | 3157 | 0.001126 | 0.7366 |
|  | 16 | rs11508026 | 56999328 | A | 3158 | -0.00279 | 0.345 |
|  | 16 | rs820299 | 57000284 | G | 3160 | 0.000761 | 0.803 |
|  | 16 | rs12708974 | 57005550 | A | 3160 | 0.006353 | 0.1534 |
|  | 16 | rs11076175 | 57006378 | G | 3155 | 0.003741 | 0.341 |
|  | 16 | rs289714 | 57007451 | G | 3140 | 0.003215 | 0.4246 |
|  | 16 | rs4784744 | 57011185 | A | 3157 | 0.0052 | 0.09703 |
|  | 16 | rs5882 | 57016092 | G | 3158 | -0.00207 | 0.5093 |
|  | 16 | rs9923854 | 57017002 | C | 3021 | -0.00307 | 0.5167 |
|  | 16 | rs289741 | 57017474 | G | 3160 | -0.00171 | 0.59 |
|  | 17 | rs6502966 | 6588586 | A | 3159 | -0.0011 | 0.7713 |
|  | 17 | rs218687 | 6588847 | A | 3160 | -0.00031 | 0.9513 |
|  | 17 | rs218688 | 6589137 | A | 3160 | -0.00186 | 0.5236 |
|  | 17 | rs218690 | 6589883 | G | 3158 | 0.003636 | 0.3374 |
|  | 17 | rs3744394 | 6591149 | A | 3160 | 0.002481 | 0.5593 |
|  | 17 | rs8069271 | 6592765 | A | 3156 | 0.000798 | 0.8538 |
|  | 17 | rs4796540 | 6594322 | A | 3159 | 0.000333 | 0.9302 |
|  | 17 | rs16956158 | 6594844 | G | 3138 | 0.001638 | 0.6189 |
|  | 17 | rs17670749 | 6596248 | A | 3156 | 0.008217 | 0.1033 |
|  | 17 | rs218669 | 6599683 | A | 3159 | 0.000634 | 0.831 |
|  | 17 | rs218673 | 6605126 | G | 3151 | -0.00307 | 0.3452 |
|  | 17 | rs9906062 | 6606748 | A | 3157 | -0.00744 | 0.0747 |
|  | 17 | rs218681 | 6613192 | A | 3159 | -0.00187 | 0.5935 |
|  | 17 | rs218682 | 6614288 | A | 3143 | -0.00118 | 0.6951 |
|  | 17 | rs1641510 | 7561496 | A | 3159 | 0.004932 | 0.09485 |
|  | 17 | rs8073498 | 7569698 | C | 3158 | -0.00227 | 0.4538 |
|  | 17 | rs12951053 | 7577407 | C | 3160 | -0.00449 | 0.4209 |
|  | 17 | rs2078486 | 7583083 | A | 3159 | 0.00137 | 0.8056 |
|  | 17 | rs8064946 | 7589311 | C | 3153 | 0.001308 | 0.7785 |
|  | 17 | rs2287499 | 7592168 | G | 3156 | 0.002519 | 0.5896 |
|  | 17 | rs2287498 | 7592560 | A | 3159 | 0.001929 | 0.7301 |
|  | 17 | rs4795893 | 32574448 | A | 3159 | -0.00421 | 0.171 |
|  | 17 | rs2857653 | 32575759 | A | 3157 | 0.001823 | 0.6174 |
|  | 17 | rs11867200 | 32575969 | A | 3160 | 0.000746 | 0.8347 |
|  | 17 | rs1024611 | 32579788 | G | 3158 | -0.00237 | 0.4797 |
|  | 17 | rs1024610 | 32580231 | A | 3160 | 0.00211 | 0.5595 |
|  | 17 | rs991804 | 32587725 | A | 3160 | -0.00268 | 0.4235 |
|  | 17 | rs4795894 | 32591444 | A | 3158 | -0.00265 | 0.4285 |
|  | 17 | rs7501939 | 36101156 | A | 3159 | 0.00399 | 0.1824 |
|  | 17 | rs3817160 | 37793815 | C | 3159 | 0.000514 | 0.8868 |
|  | 18 | rs1792689 | 45368587 | A | 3156 | -0.00804 | 0.07153 |
|  | 18 | rs1792658 | 45382605 | C | 3156 | -0.00393 | 0.2772 |
|  | 18 | rs1787177 | 45394919 | G | 3160 | -0.00488 | 0.3949 |
|  | 18 | rs1631576 | 45403483 | A | 3130 | 0.002908 | 0.3267 |
|  | 18 | rs1787199 | 45404603 | T | 3159 | -0.00409 | 0.1616 |
|  | 18 | rs729112 | 45417160 | A | 3158 | 0.002982 | 0.3129 |
|  | 18 | rs4940086 | 45446307 | G | 3158 | -0.00176 | 0.5682 |
|  | 19 | rs5030340 | 10382281 | A | 3159 | -0.00789 | 0.197 |
|  | 19 | rs5030390 | 10382537 | A | 3159 | 0.01035 | 0.05407 |
|  | 19 | rs281432 | 10390658 | G | 3158 | 0.001549 | 0.5995 |
|  | 19 | rs1799969 | 10394792 | A | 3077 | 0.000351 | 0.94 |
|  | 19 | rs5498 | 10395683 | G | 3158 | -0.00044 | 0.8848 |
|  | 19 | rs3093032 | 10396336 | A | 3156 | 0.001676 | 0.6884 |
|  | 19 | rs281438 | 10399375 | C | 3160 | 0.003474 | 0.2785 |
|  | 19 | rs281440 | 10400304 | G | 3158 | -0.0039 | 0.278 |
|  | 19 | rs2228615 | 10403368 | A | 3160 | -0.00073 | 0.81 |
|  | 19 | rs3181049 | 10441117 | A | 3150 | 0.003275 | 0.3813 |
|  | 19 | rs2278442 | 10444826 | G | 3156 | 0.002335 | 0.4514 |
|  | 19 | rs281413 | 10446734 | A | 3138 | -0.00128 | 0.7354 |
|  | 19 | rs1058154 | 10446897 | A | 3160 | 0.002461 | 0.476 |
|  | 19 | rs2304240 | 10449392 | A | 3160 | 0.003131 | 0.4297 |
|  | 19 | rs3176768 | 10449665 | A | 3154 | 0.002572 | 0.4587 |
|  | 19 | rs281414 | 10450285 | A | 3160 | 0.000782 | 0.845 |
|  | 19 | rs8109627 | 41822986 | G | 3159 | 0.001046 | 0.7551 |
|  | 19 | rs3826714 | 41825776 | G | 3157 | -0.0002 | 0.9586 |
|  | 19 | rs10417924 | 41833167 | A | 3152 | -6.73E-05 | 0.9856 |
|  | 19 | rs4803455 | 41851509 | C | 3157 | 0.003496 | 0.2313 |
|  | 19 | rs2241715 | 41856886 | A | 3159 | 0.003814 | 0.2272 |
|  | 19 | rs1982072 | 41864509 | T | 3159 | 0.003577 | 0.2567 |
|  | 19 | rs188440 | 48372133 | G | 3159 | -0.0015 | 0.6459 |
|  | 19 | rs182420 | 48372195 | G | 3158 | -0.00293 | 0.397 |
|  | 19 | rs296368 | 48372298 | G | 3158 | -0.00122 | 0.7089 |
|  | 19 | rs2910393 | 48381128 | A | 3158 | -0.00194 | 0.5526 |
|  | 19 | rs2547238 | 48382480 | G | 3159 | 0.003361 | 0.3045 |
|  | 19 | rs2547231 | 48385057 | C | 3133 | 0.0007 | 0.861 |
|  | 19 | rs212099 | 48389002 | A | 3157 | 0.000258 | 0.9487 |
|  | 19 | rs2932766 | 48390424 | C | 3157 | -0.00073 | 0.8226 |
|  | 19 | rs7508610 | 48391576 | A | 3150 | -0.00177 | 0.5877 |
|  | 19 | rs2910400 | 48394042 | C | 3157 | -0.00105 | 0.7485 |
|  | 21 | rs7278223 | 27254218 | A | 3157 | 0.002548 | 0.5475 |
|  | 21 | rs214484 | 27254279 | G | 3158 | -0.00126 | 0.6851 |
|  | 21 | rs3787620 | 27255117 | C | 3157 | -0.00662 | 0.1546 |
|  | 21 | rs2829972 | 27259699 | G | 3158 | -0.00177 | 0.5515 |
|  | 21 | rs2070653 | 27263593 | G | 3158 | -0.00307 | 0.3399 |
|  | 21 | rs380417 | 27272159 | A | 3158 | 0.0017 | 0.6485 |
|  | 21 | rs2234982 | 27277907 | A | 3155 | -0.00276 | 0.4541 |
|  | 21 | rs1782978 | 27278998 | G | 3158 | 0.004602 | 0.3749 |
|  | 21 | rs1783016 | 27280038 | A | 3150 | 0.002679 | 0.4495 |
|  | 21 | rs2829984 | 27304252 | A | 3159 | 0.003409 | 0.4662 |
|  | 21 | rs2234983 | 27316874 | A | 3159 | -0.00457 | 0.2161 |
|  | 21 | rs429410 | 27326510 | A | 3158 | -0.00461 | 0.167 |
|  | 21 | rs2829997 | 27326859 | G | 3157 | -0.00192 | 0.5436 |
|  | 21 | rs2014146 | 27335022 | G | 3158 | -0.00127 | 0.6786 |
|  | 21 | rs216762 | 27336168 | G | 3160 | 0.001661 | 0.5773 |
|  | 21 | rs9636777 | 27356669 | C | 3155 | 0.005613 | 0.1389 |
|  | 21 | rs7278838 | 27374604 | A | 3157 | 0.002602 | 0.4482 |
|  | 21 | rs928902 | 27390484 | A | 3155 | 0.001472 | 0.6756 |
|  | 21 | rs2830022 | 27411948 | C | 3158 | 0.001553 | 0.6247 |
|  | 21 | rs2830025 | 27422395 | A | 3160 | -0.00339 | 0.3451 |
|  | 21 | rs2830028 | 27427248 | A | 3159 | 0.000205 | 0.9505 |
|  | 21 | rs2830029 | 27427592 | G | 3158 | -0.00261 | 0.4135 |
|  | 21 | rs2830030 | 27429171 | A | 3160 | -0.00072 | 0.835 |
|  | 21 | rs2830038 | 27442596 | G | 3158 | -0.00163 | 0.5918 |
|  | 21 | rs17514385 | 27452642 | A | 3160 | 0.001194 | 0.8137 |
|  | 21 | rs2830045 | 27458409 | G | 3156 | 0.003914 | 0.1975 |
|  | 21 | rs2830046 | 27458706 | G | 3159 | -0.00383 | 0.241 |
|  | 21 | rs2830051 | 27465355 | G | 3160 | 0.000158 | 0.9662 |
|  | 21 | rs2830079 | 27506241 | G | 3160 | 0.005751 | 0.195 |
|  | 21 | rs466609 | 27506317 | C | 3154 | -0.00366 | 0.4233 |
|  | 21 | rs2226350 | 27516617 | G | 3155 | -0.00312 | 0.3103 |
|  | 21 | rs17588612 | 27517203 | G | 3158 | -0.00747 | 0.1321 |
|  | 21 | rs2154482 | 27520931 | A | 3160 | -0.00078 | 0.7891 |
|  | 21 | rs455465 | 27522019 | C | 3157 | 0.004389 | 0.2932 |
|  | 21 | rs4817090 | 27534261 | G | 3154 | 0.003122 | 0.3076 |
|  | 21 | rs2830101 | 27534816 | A | 3160 | -0.00383 | 0.2212 |
|  | 22 | rs737866 | 19930109 | G | 3156 | 0.000374 | 0.9089 |
|  | 22 | rs933271 | 19931407 | G | 3157 | -0.0061 | 0.06459 |
|  | 22 | rs5993883 | 19937638 | A | 3155 | 0.003966 | 0.1833 |
|  | 22 | rs6269 | 19949952 | G | 3145 | 0.003474 | 0.2464 |
|  | 22 | rs740601 | 19950763 | C | 3156 | 0.003164 | 0.29 |
|  | 22 | rs4818 | 19951207 | C | 3149 | 0.003093 | 0.3039 |
|  | 22 | rs4680 | 19951271 | G | 3115 | -0.0016 | 0.5871 |
|  | 22 | rs4646316 | 19952132 | A | 3157 | 0.002484 | 0.4672 |
|  | 22 | rs165774 | 19952561 | A | 3140 | 0.002506 | 0.4234 |
|  | 22 | rs174696 | 19953176 | G | 3157 | -0.00856 | *0.01905* |
|  | 22 | rs9332377 | 19955692 | A | 3160 | 0.002533 | 0.5262 |
|  | 22 | rs4253623 | 46550106 | G | 3158 | 0.00012 | 0.9769 |
|  | 22 | rs135539 | 46559267 | C | 3159 | 0.001134 | 0.6971 |
|  | 22 | rs4253655 | 46569171 | A | 3158 | 0.000426 | 0.9149 |
|  | 22 | rs9626736 | 46570232 | G | 3157 | 0.000164 | 0.9575 |
|  | 22 | rs4253662 | 46572744 | A | 3156 | 0.005867 | 0.2556 |
|  | 22 | rs4253701 | 46586110 | A | 3158 | -0.0036 | 0.4461 |
|  | 22 | rs12330015 | 46590278 | G | 3160 | -0.00204 | 0.6788 |
|  | 22 | rs8138102 | 46591752 | G | 3158 | -0.00516 | 0.1458 |
|  | 22 | rs11703495 | 46593336 | A | 3152 | -0.00361 | 0.468 |
|  | 22 | rs4253728 | 46610067 | A | 3159 | -0.00203 | 0.538 |
|  | 22 | rs1800206 | 46614274 | C | 3160 | 0.003522 | 0.5395 |
|  | 22 | rs6008197 | 46620416 | C | 3159 | -0.00222 | 0.5938 |
|  | 22 | rs4253765 | 46622888 | A | 3160 | -0.00319 | 0.4115 |
|  | 22 | rs4253776 | 46629479 | G | 3159 | -0.00811 | 0.08661 |
|  | 22 | rs4253778 | 46630634 | C | 3158 | -0.00302 | 0.4351 |
|  | 23 | rs5741880 | 12887416 | A | 3155 | 0.001939 | 0.7215 |
|  | 23 | rs1731478 | 12889666 | A | 3156 | -0.00801 | 0.1705 |
|  | 23 | rs179021 | 12889763 | C | 3155 | 0.001095 | 0.7862 |
|  | 23 | rs1731479 | 12890453 | A | 3154 | -0.00209 | 0.6345 |
|  | 23 | rs5743749 | 12894491 | A | 3156 | -0.00047 | 0.9422 |
|  | 23 | rs179014 | 12899765 | A | 3150 | 0.000958 | 0.8112 |
|  | 23 | rs179012 | 12901562 | A | 3155 | 0.00068 | 0.851 |
|  | 23 | rs179010 | 12902885 | A | 3151 | 0.00074 | 0.8398 |
|  | 23 | rs179009 | 12903480 | G | 3153 | -0.00018 | 0.9644 |
|  | 23 | rs179008 | 12903659 | A | 3157 | -0.00031 | 0.9386 |
|  | 23 | rs864058 | 12906030 | A | 3155 | -0.00344 | 0.5671 |
|  | 23 | rs3764880 | 12924826 | G | 3156 | -0.00556 | 0.146 |
|  | 23 | rs1548731 | 12927947 | A | 3148 | 0.005398 | 0.1518 |
|  | 23 | rs2159377 | 12937513 | A | 3145 | 0.002107 | 0.6352 |
|  | 23 | rs5744088 | 12940564 | C | 3153 | 0.008412 | 0.06211 |
|  | 23 | rs571974 | 105113632 | G | 3151 | -0.004 | 0.4376 |
|  | 23 | rs17332342 | 105132225 | G | 3158 | -0.00052 | 0.9222 |
|  | 23 | rs5916959 | 105135897 | A | 3160 | -0.00437 | 0.4564 |
|  | 23 | rs5962597 | 105151664 | A | 3159 | 0.01295 | *0.03748* |

APPENDIX 3

**References**

**1.**Bergman H, Ferrucci L, Guralnik J, Hogan DB, Hummel S, Karunananthan S, Wolfson C. Frailty: An emerging research and clinical paradigm–issues and controversies. J Gerontol A Biol Sci Med Sci 2007; 62A: 731–7.

**2.**Fried LP, Tangen CM, Walston J, Newman AB, Hirsch C, Gottdiener J, Seeman T, Tracy R, Kop WJ, Burke G, McBurnie MA; Cardiovascular Health Study Collaborative Research Group. Frailty in older adults: evidence for a phenotype. J Gerontol A Biol Sci Med Sci. 2001; 56: M146-56.

**3.**Carcaillon L, Blanco C, Alonso-Bouzón C, Alfaro-Acha A, Garcia-García FJ, Rodriguez-Mañas L. Sex differences in the association between serum levels of testosterone and frailty in an elderly population: the Toledo Study for Healthy Aging. PLoS One. 2012b; 7:e32401.

**4.**Voznesensky M, Walsh S, Dauser D, Brindisi J, Kenny AM. The association between dehydroepiandosterone and frailty in older men and women. Age Ageing. 2009; 38: 401-6.

**5.**Matsumoto AM. Andropause: clinical implications of the decline in serum testosterone levels with aging in men. J Gerontol A Biol Sci Med Sci. 2002; 57: M76-99. Review.

**6.**Gray A, Feldman HA, McKinlay JB, Longcope C. Age, disease, and changing sex hormone levels in middle-aged men: results of the Massachusetts Male Aging Study. J Clin Endocrinol Metab. 1991; 73: 1016-25.

**7.**Srinivas-Shankar U, Roberts SA, Connolly MJ, O'Connell MD, Adams JE, Oldham JA, Wu FC. Effects of testosterone on muscle strength, physical function, body composition, and quality of life in intermediate-frail and frail elderly men: a randomized, double-blind, placebo-controlled study. J Clin Endocrinol Metab. 2010; 95: 639-50.

**8.**Yen SS, Morales AJ, Khorram O. Replacement of DHEA in aging men and women. Potential remedial effects. Ann N Y Acad Sci. 1995; 774: 128-42.

**9.**Wang C, Swerdloff RS, Iranmanesh A, Dobs A, Snyder PJ, Cunningham G, Matsumoto AM, Weber T, Berman N; Testosterone Gel Study Group. Transdermal testosterone gel improves sexual function, mood, muscle strength, and body composition parameters in hypogonadal men. J Clin Endocrinol Metab. 2000; 85: 2839-53.

**10.**Baker WL, Karan S, Kenny AM. Effect of dehydroepiandrosterone on muscle strength and physical function in older adults: a systematic review. J Am Geriatr Soc. 2011; 59: 997-1002.

**11.**Khosla S, Melton LJ 3rd, Atkinson EJ, O'Fallon WM, Klee GG, Riggs BL. Relationship of serum sex steroid levels and bone turnover markers with bone mineral density in men and women: a key role for bioavailable estrogen. J Clin Endocrinol Metab. 1998; 83: 2266-74.

**12.**Ghebre MA, Hart DJ, Hakim AJ, Kato BS, Thompson V, Arden NK, Spector TD, Zhai G. Association between DHEAS and bone loss in postmenopausal women: a 15-year longitudinal population-based study. Calcif Tissue Int. 2011; 89: 295-302.

**13.**Malkin CJ, Pugh PJ, Jones RD, Kapoor D, Channer KS, Jones TH. The effect of testosterone replacement on endogenous inflammatory cytokines and lipid profiles in hypogonadal men. J Clin Endocrinol Metab. 2004; 89: 3313-8.

**14.**Straub RH, Konecna L, Hrach S, Rothe G, Kreutz M, Schölmerich J, Falk W, Lang B. Serum dehydroepiandrosterone (DHEA) and DHEA sulfate are negatively correlated with serum interleukin-6 (IL-6), and DHEA inhibits IL-6 secretion from mononuclear cells in man in vitro: possible link between endocrinosenescence and immunosenescence. J Clin Endocrinol Metab. 1998; 83: 2012-7.

**15.**Bruunsgaard H, Pedersen M, Pedersen BK. Aging and proinflammatory cytokines. Curr Opin Hematol. 2001; 8: 131-6. Review.

**16.**Hubbard RE, O'Mahony MS, Savva GM, Calver BL, Woodhouse KW. Inflammation and frailty measures in older people. J Cell Mol Med. 2009; 13: 3103-9.

**17.**Searle SD, Mitnitski A, Gahbauer EA, Gill TM, Rockwood K. A standard procedure for creating a frailty index. BMC Geriatr. 2008; 8: 24.

**18.**Tajar A, O'Connell MD, Mitnitski AB, O'Neill TW, Searle SD, Huhtaniemi IT, Finn JD, Bartfai G, Boonen S, Casanueva FF, Forti G, Giwercman A, Han TS, Kula K, Labrie F, Lean ME, Pendleton N, Punab M, Silman AJ, Vanderschueren D, Rockwood K, Wu FC; European Male Aging Study Group. Frailty in relation to variations in hormone levels of the hypothalamic-pituitary-testicular axis in older men: results from the European male aging study. J Am Geriatr Soc. 2011; 59: 814-21.

**19.**Collerton J, Martin-Ruiz C, Davies K, Hilkens CM, Isaacs J, Kolenda C, Parker C, Dunn M, Catt M, Jagger C, von Zglinicki T, Kirkwood TB. Frailty and the role of inflammation, immunosenescence and cellular ageing in the very old: cross-sectional findings from the Newcastle 85+ Study. Mech Ageing Dev. 2012; 133: 456-66.

**20.** Fishman D, Faulds G, Jeffery R, Mohamed-Ali V, Yudkin JS, Humphries S, Woo P. The effect of novel polymorphisms in the interleukin-6 (IL-6) gene on IL-6 transcription and plasma IL-6 levels, and an association with systemic-onset juvenile chronic arthritis. J Clin Invest. 1998;102:1369-76.

**21.** Wilson AG, Symons JA, McDowell TL, McDevitt HO, Duff GW. [Effects of a polymorphism in the human tumor necrosis factor alpha promoter on transcriptional activation.](http://www.ncbi.nlm.nih.gov/pubmed/9096369) Proc Natl Acad Sci U S A. 1997; 94: 3195-9.

**22.**Steptoe A, Breeze E, Banks J, Nazroo J. [Cohort profile: the English Longitudinal Study of Ageing.](http://www.ncbi.nlm.nih.gov/pubmed/23143611) Int J Epidemiol. 2013; 42: 1640-8.

**23.**Purcell S, Neale B, Todd-Brown K, Thomas L, Ferreira MA, Bender D, Maller J, Sklar P, de Bakker PI, Daly MJ, Sham PC. PLINK: a tool set for whole-genome association and population-based linkage analyses. Am J Hum Genet. 2007; 81: 559-75.

**24.** Biet F, Locht C, Kremer L. Immunoregulatory functions of interleukin 18 and its role in defense against bacterial pathogens. J Mol Med (Berl). 2002;80:147-62.

**25.**Chen M, Geng JG. P-selectin mediates adhesion of leukocytes, platelets, and cancer cells in inflammation, thrombosis, and cancer growth and metastasis. Arch Immunol Ther Exp (Warsz). 2006; 54: 75-84. Review.

**26.**Ferrucci L, Corsi A, Lauretani F, Bandinelli S, Bartali B, Taub DD, Guralnik JM, Longo DL. The origins of age-related proinflammatory state. Blood. 2005; 105: 2294-9.

**27.**Frayling TM, Rafiq S, Murray A, Hurst AJ, Weedon MN, Henley W, Bandinelli S, Corsi AM, Ferrucci L, Guralnik JM, Wallace RB, Melzer D. An interleukin-18 polymorphism is associated with reduced serum concentrations and better physical functioning in older people. J Gerontol A Biol Sci Med Sci. 2007; 62: 73-8.

**28.**Oda K, Miyatake N, Sakano N, Saito T, Miyachi M, Tabata I, Numata T. Serum interleukin-18 levels are associated with physical activity in Japanese men. PLoS One. 2013; 8: e81497.

**29.**Sugiura T, Kawaguchi Y, Ikari K, Ichida H, Kawamoto M, Momohara S, Hara M, Yamanaka H. Interleukin-18 promoter polymorphisms in Japanese patients with rheumatoid arthritis: protective effect of the T allele and T/T genotype at rs360722. Mod Rheumatol. 2011; 21: 359-64.

**30.**Hirschfield GM, Liu X, Xu C, Lu Y, Xie G, Lu Y, Gu X, Walker EJ, Jing K, Juran BD, Mason AL, Myers RP, Peltekian KM, Ghent CN, Coltescu C, Atkinson EJ, Heathcote EJ, Lazaridis KN, Amos CI, Siminovitch KA. Primary biliary cirrhosis associated with HLA, IL12A, and IL12RB2 variants. N Engl J Med. 2009; 360: 2544-55.

**31.**Mathew JP, Podgoreanu MV, Grocott HP, White WD, Morris RW, Stafford-Smith M, Mackensen GB, Rinder CS, Blumenthal JA, Schwinn DA, Newman MF; PEGASUS Investigative Team. Genetic variants in P-selectin and C-reactive protein influence susceptibility to cognitive decline after cardiac surgery. J Am Coll Cardiol. 2007; 49: 1934-42.

**32.**Ovsyannikova IG, Haralambieva IH, Kennedy RB, Pankratz VS, Vierkant RA, Jacobson RM, Poland GA. Impact of cytokine and cytokine receptor gene polymorphisms on cellular immunity after smallpox vaccination. Gene. 2012; 510: 59-65.

**33.**Weyrich AS, McIntyre TM, McEver RP, Prescott SM, Zimmerman GA. Monocyte tethering by P-selectin regulates monocyte chemotactic protein-1 and tumor necrosis factor-alpha secretion. Signal integration and NF-kappa B translocation. J Clin Invest. 1995; 95: 2297-303.

**34.**Lillis AP, Van Duyn LB, Murphy-Ullrich JE, Strickland DK. LDL receptor-related protein 1: unique tissue-specific functions revealed by selective gene knockout studies. Physiol Rev. 2008; 88: 887-918.

**35.**Kang DE, Pietrzik CU, Baum L, Chevallier N, Merriam DE, Kounnas MZ, Wagner SL, Troncoso JC, Kawas CH, Katzman R, Koo EH. Modulation of amyloid beta-protein clearance and Alzheimer's disease susceptibility by the LDL receptor-related protein pathway. J Clin Invest. 2000; 106:1159-66.

**36.**Hollenbach E, Ackermann S, Hyman BT, Rebeck GW. [Confirmation of an association between a polymorphism in exon 3 of the low-density lipoprotein receptor-related protein gene and Alzheimer's disease.](http://www.ncbi.nlm.nih.gov/pubmed/9633759) Neurology. 1998; 50: 1905-7.

**37.**Lambert JC, Wavrant-De Vrièze F, Amouyel P, Chartier-Harlin MC. Association at LRP gene locus with sporadic late-onset Alzheimer's disease. Lancet. 1998; 351: 1787-8.

**38.**Chalmers KA, Barker R, Passmore PA, Panza F, Seripa D, Solfrizzi V, Love S, Prince JA, Kehoe PG. LRP-1 variation is not associated with risk of Alzheimer's disease. Int J Mol Epidemiol Genet. 2010; 1: 104-13.

**39.**Aledo R, Alonso R, Mata P, Llorente-Cortés V, Padró T, Badimon L. LRP1 gene polymorphisms are associated with premature risk of cardiovascular disease in patients with familial hypercholesterolemia. Rev Esp Cardiol (Engl Ed). 2012; 65: 807-12.
